# Otoprotective effect of MnTBAP in cisplatin-induced hearing loss

**DOI:** 10.1371/journal.pone.0351911

**Published:** 2026-09-21

**Authors:** Shomaila Mehmood, Pankaj Bhatia, Samson Jamesdaniel

**Affiliations:** 1 Institute of Environmental Health Sciences, Wayne State University, Detroit, Michigan, United States of America; 2 Department of Family Medicine and Public Health Sciences, Wayne State University, Detroit, Michigan, United States of America; University of Nis Faculty of Medicine: Univerzitet u Nisu Medicinski Fakultet, SERBIA

## Abstract

**Objective:**

Cisplatin, a life-saving chemotherapeutic drug, causes ototoxicity. Although sodium thiosulfate is used to prevent ototoxicity in pediatric patients, no other intervention has been approved for clinical use against cisplatin-induced hearing loss. Hence, there is an urgent need to identify drugs that prevent cisplatin ototoxicity.

**Methods:**

CBA/J mice were treated with cisplatin (3 mg/kg, i.p., daily for 5 days), and MnTBAP (10 mg/kg, i.p., daily for 8 days) was used to inhibit cisplatin-induced ototoxicity. Auditory brainstem responses (ABRs) and distortion product otoacoustic emissions (DPOAEs) were recorded before and after treatment to assess hearing loss, while immunohistochemistry with anti-Myosin-VIIa was conducted to examine hair cell loss, with phalloidin to examine spiral ganglion neuron (SGN) loss, and with anti-nitrotyrosine to assess nitrotyrosine levels.

**Results:**

Cisplatin treatment elevated the nitrotyrosine levels in hair cells and SGNs and increased the loss of these cells in the middle and basal cochlear regions. A negative correlation was observed between cisplatin-induced changes in the hair cell count or SGN density and nitrotyrosine levels. Cisplatin treatment elevated the hearing thresholds, as indicated by the ABRs, and lowered the DPOAE amplitudes, suggesting cisplatin-induced hearing loss. However, MnTBAP cotreatment prevented the cisplatin-induced changes in hearing sensitivity and reversed the morphological changes.

**Conclusion:**

The otoprotection observed with MnTBAP cotreatment along with the attenuation of cisplatin-induced increase in nitrotyrosine levels in the cochlea indicates its potential as an inhibitor of cochlear nitrative stress and its utility in mitigating cisplatin-induced ototoxicity.

## Introduction

Cisplatin is a highly used, efficient anti-cancer treatment against a wide range of solid tumors [1–4]. However, it has significant adverse effects, some of which might impair a patient's quality of life permanently after treatment. Particularly, cisplatin treatment causes hearing loss in approximately 60% of patients, including both pediatric and adult cancer populations, which affects their quality of life [5–7]. Both children and adults develop high-frequency hearing loss after cisplatin treatment, which can progress to cause considerable damage in frequency regions essential to understand human language [8–10]. Cisplatin-induced ototoxicity can result in mild to severe hearing impairment and is influenced by age, concurrent cranial irradiation, pre-existing hearing loss, dosing schedules, and cumulative dose [11,12]. Studies have indicated that the three primary targets of cisplatin in the cochlea are the sensory hair cells in the organ of Corti [13], the lateral wall tissues [14], and the spiral ganglion neurons (SGNs) in the modiolus [15]. Cisplatin treatment causes the death of outer hair cells (OHCs) in the basal turn of the cochlea; therefore, it affects high-frequency hearing more than low-frequency hearing [16,17]. Though the cytotoxic mechanism underlying cisplatin-induced ototoxicity is not fully understood, various studies have reported that cisplatin-induced cochlear apoptosis is intricately linked to oxidative stress, DNA damage, and inflammatory factors [11,18–20]. Moreover, among the cochlear cytotoxic mechanisms, oxidative stress plays a major role, as cisplatin activates the NOX3 enzyme, increasing the generation of superoxide radicals in the inner ear [11,21]. Additionally, the iNOS pathway is activated, leading to nitric oxide (NO) generation during cisplatin-induced ototoxicity [22,23]. The reaction of NO with superoxide radicals produces peroxynitrite (ONOO^-^) that ultimately causes protein nitration. Nitrated proteins can alter the signaling pathways that regulate apoptosis and cause damage to hair cells in the cochlea [24]. Nitration of LMO4, a transcriptional regulator involved in cell survival/death and inner ear development [25–29], has been implicated as a key mediator of cisplatin ototoxicity [24]. Thus, nitrative stress has emerged as a critical factor in cisplatin-induced hearing loss.

To mitigate cisplatin ototoxicity, numerous therapeutic strategies have been employed, among which reducing the pro-inflammatory cytokine release has shown efficacy [30–33]. Other approaches, such as the use of antioxidants sodium thiosulfate (STS) and N-acetylcysteine (NAC), reduce the intracellular reactive oxygen species (ROS) accumulation to attenuate cisplatin-induced hearing loss [34–38]. However, STS and NAC can interfere with the anti-cancer activity of cisplatin [39,40] and compromise the chemoprotection [40,41]. Therefore, it is important to identify novel interventional drugs that are highly effective in mitigating cisplatin-induced ototoxicity without interfering with its anticancer efficacy [31,42–44].

Nitrative stress has emerged as a promising target for preventing cisplatin ototoxicity. Metalloporphyrins such as FeTMPyP and MnTBAP chloride **(**Mn (III)tetrakis (4-benzoic acid) porphyrin chloride) are efficient ONOO^-^ scavengers [45], which probably contributes to their efficacy in attenuating cisplatin-induced apoptosis, nitrative stress, and nephrotoxicity [46]. They also prevent meningitis-related hearing loss resulting from increased reactive oxygen species (ROS) and ONOO^-^ [47]. Other PNDCs, such as SRI110, a Mn (III) complex, have been reported to prevent cisplatin-induced cytotoxicity and nitrative stress [48]. MnTBAP is widely used *in vivo* models of oxidative stress injuries and has been reported to selectively target ONOO^-^ when it is in a pure form [45]. As MnTBAP has been reported to inhibit both superoxide and ONOO^-^ and targeting of ONOO^-^ by metalloporphyrins is emerging as an efficient interventional strategy to mitigate cisplatin ototoxicity, we assessed the efficacy of MnTBAP cotreatment against cisplatin ototoxicity by evaluating the effect of MnTBAP cotreatment on hair cells, SGNs, and auditory function using a mouse model.

## Materials and methods

### Animals

A total of 12 male and 12 female CBA/J mice (six-week-old), obtained from Jackson Labs (Bar Harbor, ME, USA), were kept in a temperature-controlled room with 12-hour light/dark cycles in the laboratory animal facility. All animal procedures, including defined humane endpoints and euthanasia criteria, received approval from the Institutional Animal Care and Use Committee (IACUC) at Wayne State University (IACUC-23-07-5961). All the experiments were conducted in accordance with applicable guidelines, regulations, and following the ARRIVE 2.0 Essential 10 guidelines. All animals were allowed a week to acclimatize. Personnel involved in animal handling and experimentation received training approved by the Wayne State University Division of Laboratory Animal Resources (DLAR), including instruction in rodent handling, recognition of pain and distress, and humane euthanasia procedures. Animals were monitored twice daily (morning and evening) throughout the study for signs of morbidity, pain, or distress, including hunched posture, piloerection, lethargy, abnormal vocalization, and reduced mobility. Body weight was recorded daily as an indicator of overall health status and as a criterion for humane intervention. Humane endpoint criteria included body weight loss exceeding 20% of baseline body weight or evidence of severe, unremitting pain or distress, such as inability to ambulate, inability to access food or water, or lack of response to gentle stimuli. No animals died during the treatment period or reached humane endpoint criteria. To minimize treatment-related morbidity, all animals received supportive care consisting of daily subcutaneous administration of 1 mL sterile 0.9% saline to mitigate dehydration associated with cisplatin treatment. At the end of the study period (8^th^ day), all the animals were euthanized by CO_2_ inhalation followed by decapitation using a guillotine, adhering to AVMA recommendations for euthanasia, and the cochlear tissue was collected for analysis.

### Drug administration

**Fig 1:**
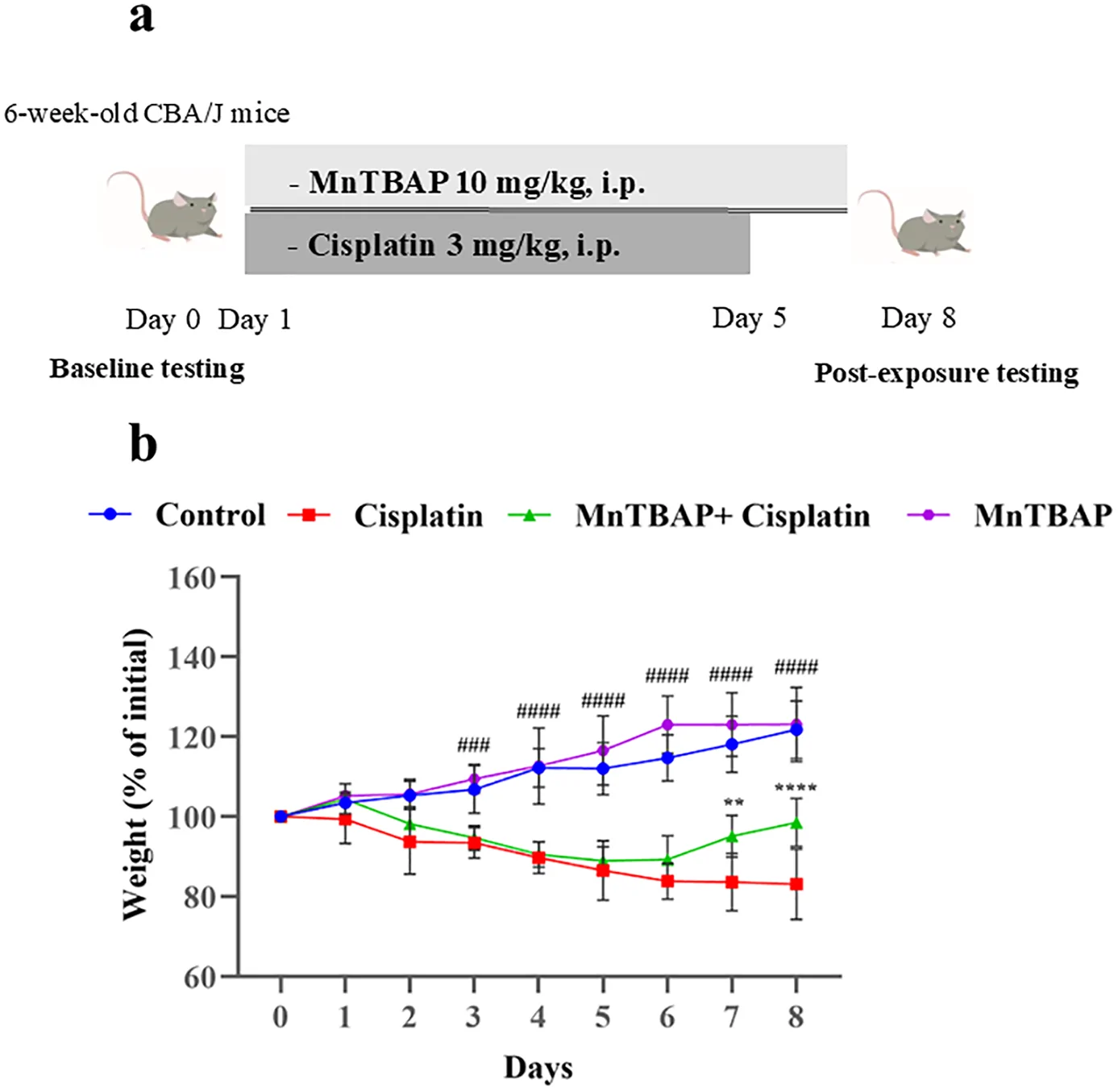
Experimental protocol and weight monitoring. (a) Cisplatin was administered for 5 days (3 mg/kg, i.p.), MnTBAP cotreatment (10 mg/kg, i.p.) was given for 5 days, 3 hours before cisplatin injection, followed by MnTBAP alone for 3 days. MnTBAP alone group received MnTBAP at the same dosage. Auditory testing was performed before the treatment began and on day 8 after the completion of the treatment protocol. Mice were given 1 mL sterile saline every day for 8 days to avoid dehydration. (b) Weight change over the 8 days was monitored for animals in all groups. MnTBAP cotreatment prevented cisplatin-induced weight loss. ^###^indicates p < 0.001, ^####^p < 0.0001 for Cisplatin vs Control and ^**^indicates p < 0.01, ^****^p < 0.0001 for MnTBAP+Cisplatin vs Cisplatin. Control animals were treated with a saline solution. Data are expressed as mean±S.D; n = 6 (3 male+3 female).

The animals were treated with cisplatin following an 8-day protocol (Fig 1). Mice were assigned to one of four experimental groups: control, cisplatin, MnTBAP+Cisplatin, and MnTBAP alone, maintaining a balance of males and females in each group. Animals in the cisplatin treatment group received a gradual intraperitoneal (i.p.) infusion of veterinary-grade cisplatin (Accord Healthcare, NDC16729-288-38, USA) diluted in sterile saline (pH 7.4) at a dose of 3 mg/kg body weight each day for 5 days. This dose was chosen based on previous studies, as it resulted in significant hearing loss [48]. The animals in the MnTBAP cotreatment group received i.p. injections of 10 mg/kg of MnTBAP (Lot # 10524, Catalog no. sc-221954A, Santa Cruz Biotechnology) each day. The purity of the MnTBAP was determined as ≥ 97% by mass spectrometry analysis. MnTBAP was dissolved in sterile saline and administered 3 hours before cisplatin injections for 5 days, followed by MnTBAP treatment alone for the remaining 3 days. The MnTBAP alone group received MnTBAP on all 8 days, while an equivalent volume of saline was administered to the control animals. All animals were subcutaneously hydrated with 1 mL of 0.9% saline every day.

### Hearing tests

Hearing was tested in all mice by recording auditory brainstem responses (ABR) and distortion product otoacoustic emissions (DPOAEs), before and after drug administration. Auditory testing was conducted using Tucker Davis Technologies (TDT) System III hardware, and sound stimuli were presented using the BioSigRZ software (v5.7.6.). Mice were put on a temperature-controlled heating pad kept at 38 °C within the sound-proof chamber (ECKEL-AB-4230, Morrisburg, Ontario) after being anesthetized with isoflurane (4% induction, 1.5% maintenance with 1 L/min O_2_). For ABR recordings, broadband click stimuli (90−20 dB SPL, 5 dB intervals) and tone bursts at 4, 8, 16, 24, and 32 kHz frequencies (90−25 dB SPL, 5 dB intervals) were delivered. To obtain the ABR waveforms, 512 stimulus presentations delivered at 21 Hz were averaged. The hearing threshold was recorded as the lowest stimulation level at which a waveform with a discernible peak was observed.

Two primary tones, f1 and f2, were used to assess DPOAEs at a f2/f1 ratio of 1.2. For L1 levels ranging from 80 dB SPL to 20 dB SPL in 10 dB increments, the L2–L1 was maintained at +10 dB. The stimulus was produced using the Tucker-Davis Technology (TDT) RZ6 system, and the multifield magnetic speakers (TDT, Alachua, FL, USA) were used to provide f1 and f2. The f2 frequency ranged between 4 and 40 kHz. The ER10B+probe microphone (Etymotic Research, Inc., Elk Grove Village, IL, USA) and TDT hardware and software were used to measure SPLs at the cubic difference frequency (2f1–f2). Distortion product data were collected every 20.971 ms and averaged 512 times, while a noise floor was measured using a 100 kHz band around 2f1–f2.

### Immunohistochemistry

Following euthanasia, the temporal bones were promptly removed in ice-cold PBS, and the blood in the cochlea was flushed out by perfusing it with 4% paraformaldehyde (PFA) in 1x PBS (pH 7.4) through the oval window. The cochlea was decalcified using 100 mM ethylenediaminetetraacetic acid disodium salt (EDTA) solution in 1x PBS (pH 7.4) for almost 48–72 hours. After decalcification, the tissues were microdissected under a stereomicroscope (Leica model # M 165 C) in ice-cold PBS. The cochlear sensory epithelium was separated from the lateral wall and bone capsule and was divided into apex, middle, and basal parts. Each cochlear section was initially blocked with 10% normal goat serum (NGS) for 1 hour at room temperature and washed thrice with 1x PBS (pH 7.4). Following this, the cochleae were incubated in two sets of primary and secondary antibodies. The first set included mouse monoclonal Myosin-VIIa (Santa Cruz Biotechnology, catalog no. sc-74516, 1:500) and goat anti-mouse Alexa Fluor^TM^ 488 (Thermo Fisher Scientific, catalog no. 21131, 1:500) while the second set consisted of mouse monoclonal nitrotyrosine (Santa Cruz Biotechnology catalog no. sc-32757, 1:400) and goat anti-mouse Alexa Fluor^TM^ 568 (Thermo Fisher Scientific, catalog no. 21124, 1:500). The tissue sections were incubated in the primary antibodies overnight at 4 °C followed by incubation with secondary antibodies for 2 hours at room temperature. The specimen were further incubated with Alexa Fluor^TM^ 488 Phalloidin (Invitrogen^TM^, Thermo Fischer Scientific, Catalog no. A12379, 1:40) for 30 minutes at room temperature and then mounted with ProLong^TM^ Gold antifade with DAPI (Invitrogen^TM^, Thermo Fischer Scientific, catalog no. P36935). The Biotium CoverGrip^TM^ Coverslip Sealant (Thermo Fisher Scientific, catalog no. NC0154994) was used to seal the coverslips.

### Confocal microscopy and image analyses

High-resolution confocal images were collected with Carl Zeiss Laser Scanning Systems (Zeiss LSM-800) using an oil-immersion 40 × objective. The hair cells in the control and drug-treated groups were counted using myosin-VIIa staining in 50 µm cochlear sections [49]. The fluorescence intensity of nitrotyrosine staining was measured in 12–14 OHCs [50] and 3 SGNs per image. The SGN density per 10^4^ µm^2^ was calculated following the protocol used by Hu et al. (2021) using ImageJ/Fiji software (v 1.46r) [51].

### Statistical analyses

All the experiments were performed in 5–6 biological replicates. Both male and female mice were used in all experiments and analyzed together. Sex was not considered as a biological variable because the number of animals in each sex was inadequate to analyze them separately. Data generated from the left ear only were analyzed for all outcomes; bilateral measurements were not included in the analysis. A two-way analysis of variance (ANOVA) test followed by Tukey’s multiple comparisons test was used for statistical analyses, and a p-value of < 0.05 was considered statistically significant. The association between cisplatin-induced changes in nitrotyrosine levels and hair cell count and SGN density was determined using Pearson’s correlation coefficient at 95% confidence interval. All data were analyzed using GraphPad Prism software (v10.2.3, La Jolla, CA, USA) and expressed as mean ± standard deviation (S.D) or mean ± standard error mean (S.E.M).

## Results

### MnTBAP cotreatment prevented cisplatin-induced weight loss

Body weight was monitored daily throughout the 8-day treatment protocol (Fig 1a) to assess the systemic effects of cisplatin and the potential protective role of MnTBAP. Mice receiving cisplatin alone exhibited a progressive decline in body weight starting from day 2, reaching a significant reduction on day 8 compared to control animals. In contrast, mice cotreated with MnTBAP exhibited significantly less weight loss, with body weight on day 8 remaining substantially higher than that of the cisplatin-treated group. The MnTBAP alone group maintained body weights comparable to controls throughout the study period, indicating MnTBAP treatment alone did not alter the body weight of animals (Fig 1b). These results suggest that MnTBAP cotreatment probably reduces cisplatin-induced systemic toxicity, although evaluation of biochemical markers of liver and renal function could further demonstrate the safety of this compound.

### Cisplatin treatment increased nitrotyrosine levels in hair cells and SGNs

**Fig 2:**
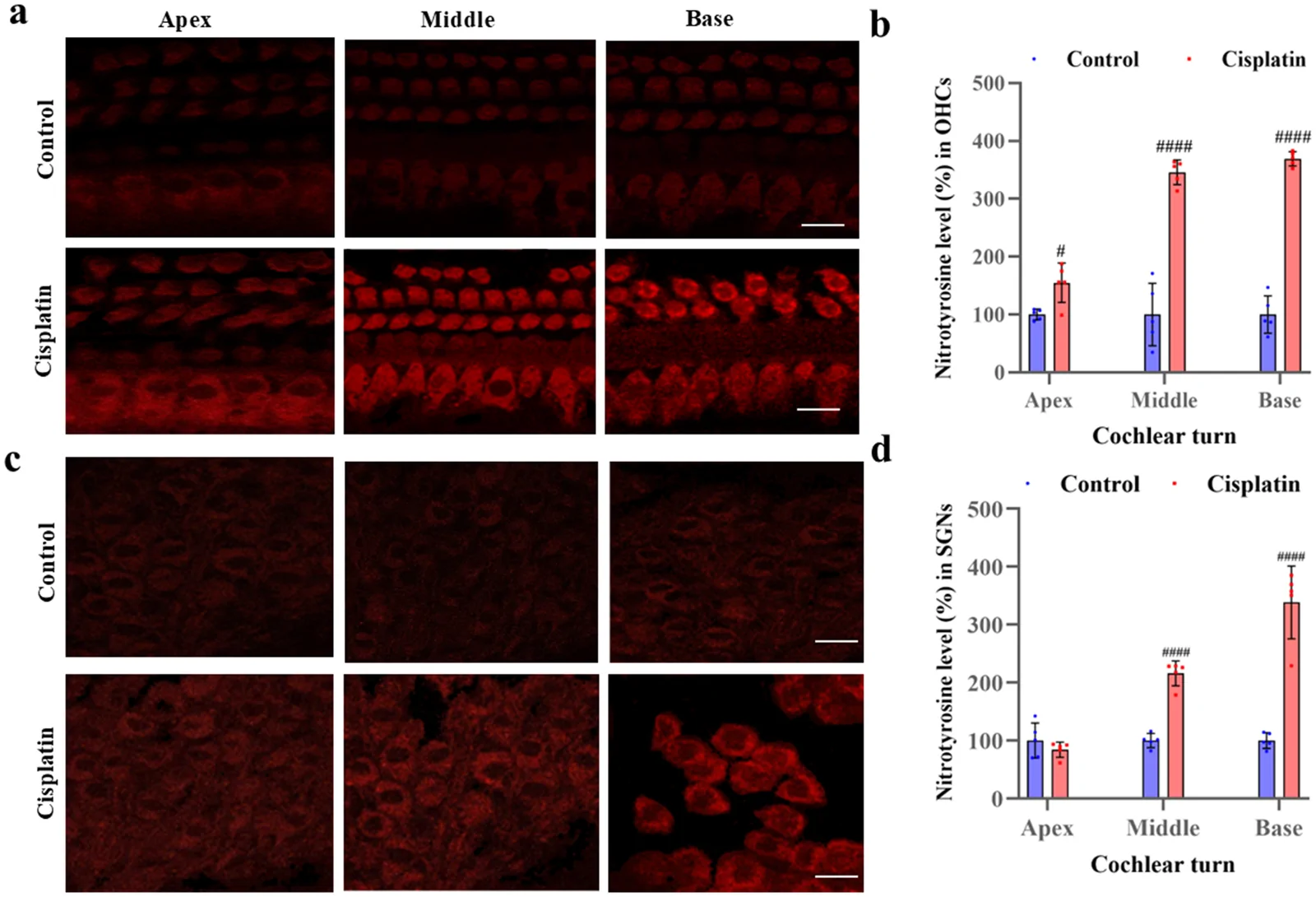
Nitrotyrosine levels in hair cells and SGNs. (a) Immunohistochemistry with nitrotyrosine antibody showed that cisplatin treatment increased nitrotyrosine levels (red) in OHCs in the apex, middle, and basal turns as compared to controls. (b) Quantification of nitrotyrosine staining intensity indicated that cisplatin significantly increased the nitrotyrosine levels across all three cochlear regions. (c) Cisplatin treatment increased nitrotyrosine levels in the SGNs in the apex, middle, and basal turns of the cochlea. (d) Quantification of nitrotyrosine staining intensity indicated a significant increase in nitrotyrosine levels in the middle and basal SGNs in the cisplatin-treated cochlea. ^#^indicates p < 0.05, ^####^p < 0.0001 for Cisplatin vs Control. Saline-treated mice were used as controls. Data from the left ear are expressed as mean±S.D; n = 5 (3 male+2 female). Scale bar = 20 μm. OHCs, outer hair cells; SGNs, spiral ganglion neurons.

Immunohistochemistry of the apex, middle and basal cochlear turns with anti-nitrotyrosine indicated that cisplatin treatment increased the nitrotyrosine levels in OHCs (Fig 2a). The quantification of nitrotyrosine staining intensity indicated that cisplatin caused a significant increase in nitrotyrosine levels across all the three regions of the cochlea as compared to the control (Fig 2b). Likewise, cisplatin treatment also increased the nitrotyrosine levels in the SGNs in the middle and basal cochlear turns, compared to the controls (Fig 2c and d). These results indicate that cisplatin treatment induces nitrative stress in the cochlea.

### MnTBAP cotreatment mitigated cisplatin-induced OHC loss

**Fig 3:**
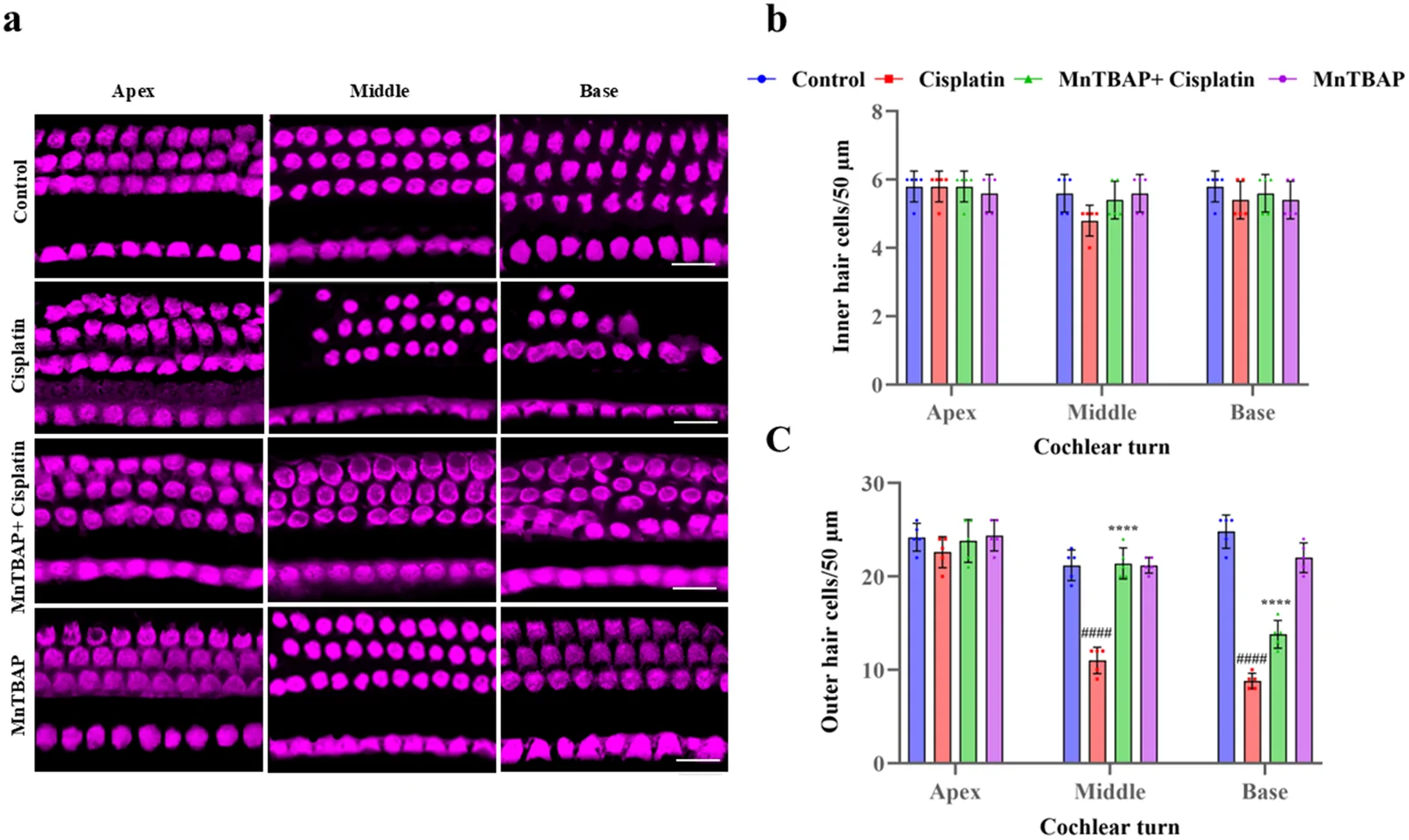
Effect of MnTBAP cotreatment on cisplatin-induced OHC loss. (a) Immunohistochemistry with anti-Myosin-VIIa indicated a significant loss of OHCs in cisplatin-treated mice in the middle and basal turns of the cochlea compared with controls. However, MnTBAP cotreatment significantly mitigated cisplatin-induced OHCs loss. (b) and (c) show the numbers of IHCs and OHCs in all three cochlear turns. These graphs illustrate significant loss of OHCs in cisplatin-treated mice in the middle and basal regions (c) and no loss of IHCs (b). ^####^ indicates p < 0.0001 for Cisplatin vs Control and ^****^ indicates p < 0.0001 for MnTBAP+Cisplatin vs Cisplatin. Saline-treated mice were used as controls. Data from the left ear are expressed as mean±S.D; n = 5 (3 male+2 female). Scale bar = 10 µm. IHC, inner hair cells; OHCs, outer hair cells.

Immunohistochemical analysis with anti-Myosin-VIIa was used to assess the hair cell loss in three cochlear regions across all four groups (Fig 3a). This analysis indicated that cisplatin treatment resulted in OHC loss, particularly in the middle and basal regions, as compared to the control and MnTBAP alone groups. There was no loss of inner hair cells (IHCs) in all groups. However, MnTBAP cotreatment prevented the cisplatin-induced loss of OHCs in the middle and basal turns. The count of IHCs and OHCs (Fig 3b and c) indicated that MnTBAP cotreatment mitigates cisplatin ototoxicity by preventing the death of OHCs after cisplatin treatment.

### MnTBAP cotreatment mitigated cisplatin-induced SGN loss

**Fig 4:**
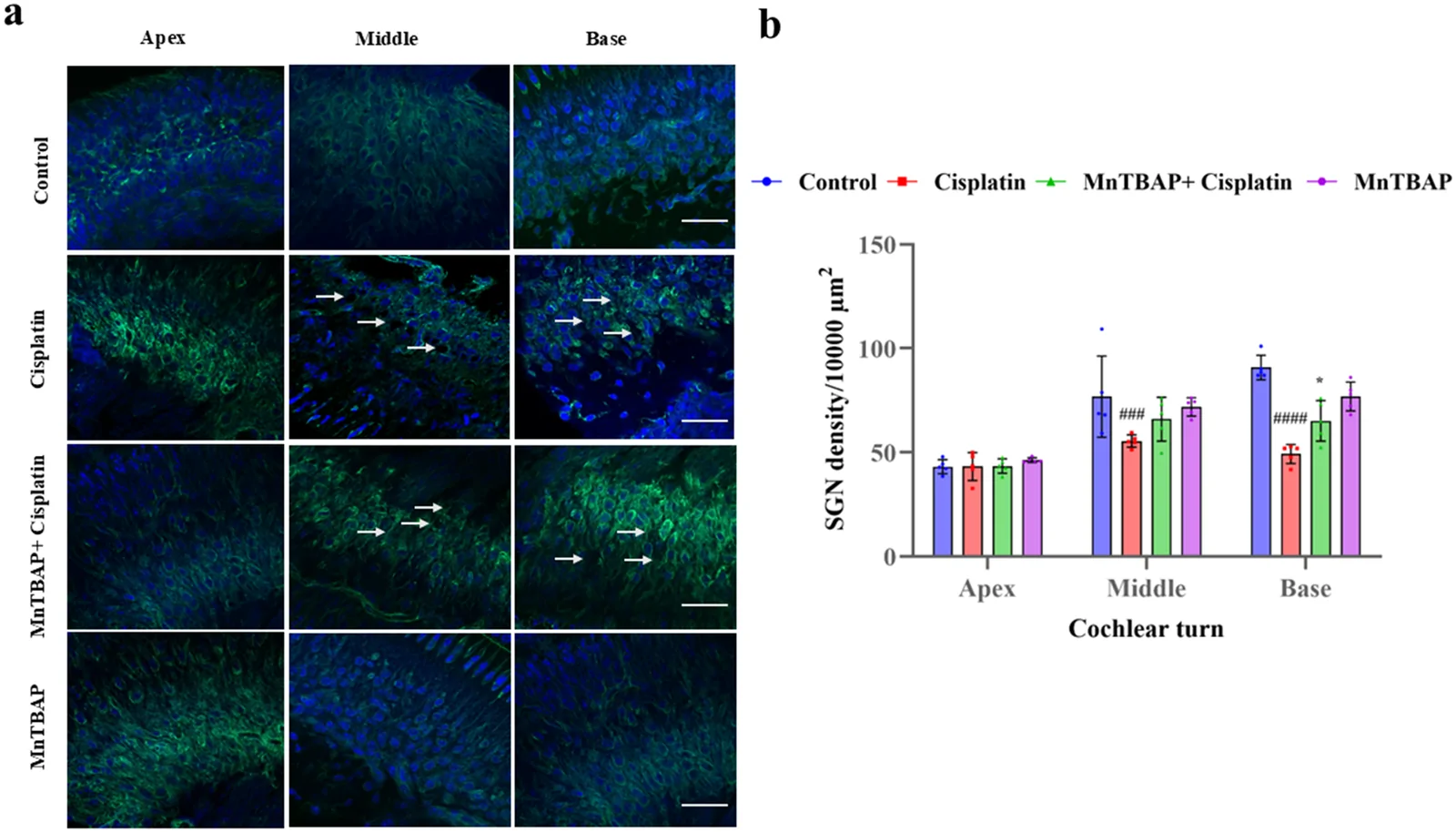
Effect of MnTBAP cotreatment on cisplatin-induced SGN loss (a) Cisplatin treatment resulted in the loss of SGNs in the middle and basal turns of the cochlea, as shown by the phalloidin (green) and DAPI (blue) staining. However, MnTBAP cotreatment prevented the cisplatin-induced SGN loss in the middle and basal turns of the cochlea. (b) The quantification of SGN density indicated that cisplatin treatment significantly decreased the SGN density, which was reversed by MnTBAP cotreatment. ^###^indicates p < 0.001, ^####^p < 0.0001 for Cisplatin vs Control and ^*^indicates p < 0.05 for MnTBAP+Cisplatin vs Cisplatin. Saline-treated mice were used as controls. Data from the left ear are expressed as mean±S.D., n = 5 (3 male+2 female). Scale bar = 20 µm. SGNs, spiral ganglion neurons.

The SGNs were visualized by staining actin with phalloidin, which indicated that cisplatin treatment significantly reduced the SGN density in the middle and basal regions of the cochlea. However, MnTBAP cotreatment significantly attenuated the cisplatin-induced decrease in the SGNs density in the middle and basal cochlear regions (Fig 4a). The quantification of SGN density revealed a higher SGN loss in the middle and basal turns in the cisplatin-treated cochlea, which was significantly reversed by the MnTBAP cotreatment, particularly in the basal turn (Fig 4b). This indicates that MnTBAP prevents cisplatin-induced SGN loss in the cochlea.

### Cisplatin-induced changes in nitrotyrosine level correlated with OHC and SGN loss

**Fig 5:**
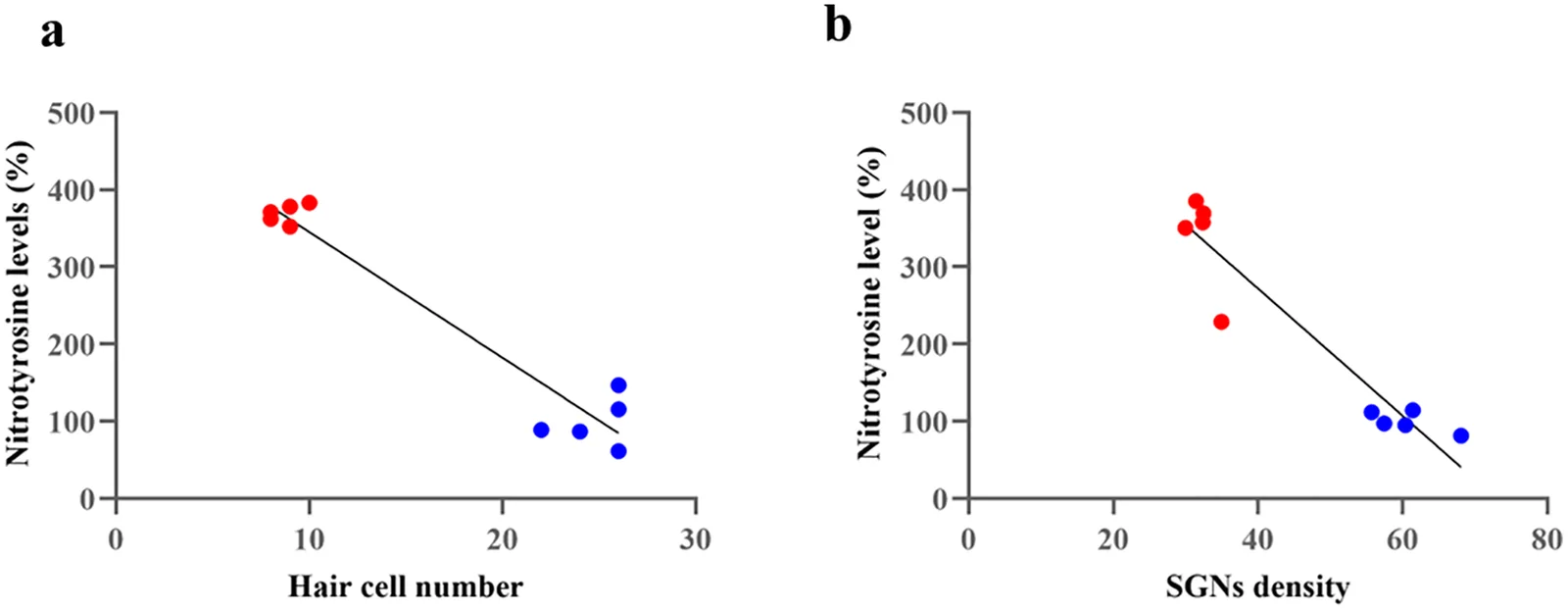
Correlation between nitrotyrosine levels and cisplatin-induced hair cell and SGN loss. Cisplatin-induced increase in nitrotyrosine levels showed a strong correlation with changes in hair cell count and SGN density. (a) and (b) Analysis of correlation between cisplatin-induced changes in the OHC count, SGNs density, and nitrotyrosine levels indicated a negative correlation (R^2^ = 0.9353 and R^2^ = 0.9069, respectively with p < 0.001; n = 5 (3 male+2 female). OHC, outer hair cell; SGNs, spiral ganglion neurons.

The correlation analysis between cisplatin-induced changes in nitrotyrosine levels and hair cell count using simple linear regression model indicated a strong negative correlation between the two variables (R^2^ = 0.9353, Fig 5a). Similarly, nitrotyrosine levels and SGNs density also revealed a negative correlation (R^2^ = 0.9069, Fig 5b). These observations are in agreement with our previous studies and indicate that higher nitrotyrosine levels after cisplatin treatment could potentially contribute to OHC and SGN death in the cochlea. As the number of replicates in this analysis is small (n = 5), these findings should be verified in follow-up studies with a larger sample size.

### MnTBAP cotreatment attenuated cisplatin-induced hearing loss

**Fig 6:**
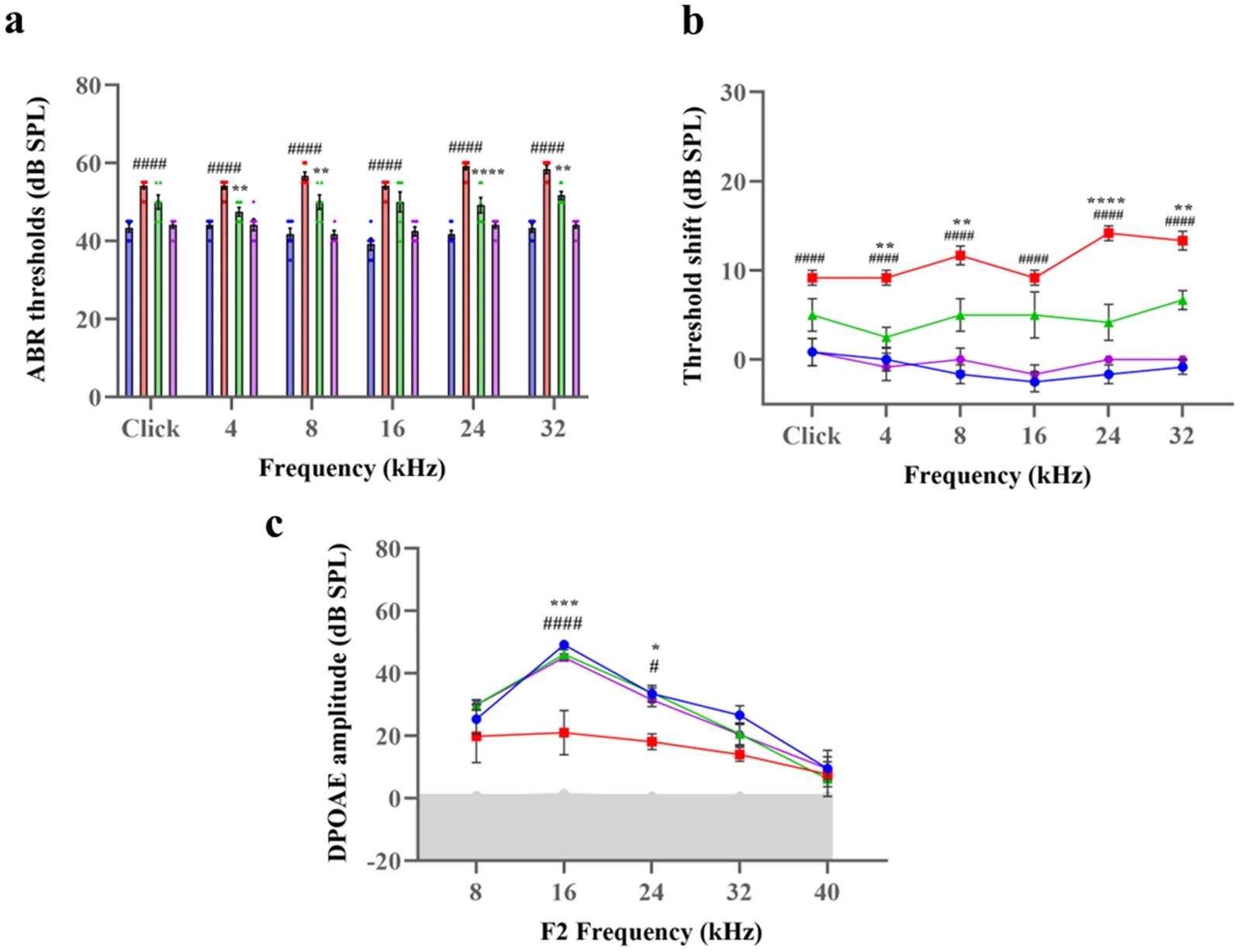
Effect of MnTBAP cotreatment on cisplatin-induced hearing loss. (a) The ABRs indicated that cisplatin-treated mice showed significantly higher hearing thresholds than control mice at multiple frequencies. However, MnTBAP cotreatment significantly attenuated the cisplatin-induced increase in hearing thresholds at 4, 8, 24, and 32 kHz. (b) Cisplatin-treated mice showed substantially higher threshold shifts than control mice; however, MnTBAP cotreatment significantly attenuated the cisplatin-induced hearing threshold shifts. (c) The DPOAEs recorded using 8, 16, 24, 32, and 40 kHz f2 stimuli indicated that the DPOAE amplitudes elicited using 70 dB SPL (L1) stimuli were significantly decreased in cisplatin-treated mice at 16 and 24 kHz frequencies. However, MnTBAP cotreatment significantly attenuated the cisplatin-induced decrease in DPOAEs amplitude at 16 and 24 kHz. Gray shaded region indicates the mean biological noise floor. ^#^ indicates p < 0.05, ^####^p < 0.0001 for Cisplatin vs Control and ^*^ indicates p < 0.05, ^**^p < 0.01, ^***^p < 0.001, ^****^p < 0.0001 for MnTBAP+Cisplatin vs Cisplatin. Saline-treated mice were used as controls. Data from the left ear are expressed as mean±S.E.M., n = 6 (3 male+3 female for ABR thresholds and threshold shift) and n = 5-6 (3 male+3 female for DPOAEs: Control, Cisplatin, MnTBAP and 3 male+2 female for MnTBAP+Cisplatin). ABR, auditory brainstem response; DPOAE, distortion product otoacoustic emissions.

Auditory sensitivity was assessed using ABR recordings after cisplatin exposure, which indicated that cisplatin treatment induced a significant increase in the ABR thresholds with threshold shifts ranging from 9 to 14 dB. However, cotreatment with MnTBAP, attenuated the cisplatin-induced increase in ABR thresholds and threshold shifts (Fig 6a, b). Similarly, cochlear OHC function was assessed using DPOAEs, which showed a significant decrease in DPOAE amplitudes in cisplatin-treated mice (Fig 6c). The DPOAE amplitudes were significantly lower in the mid-high frequency regions (16 and 24 kHz). However, MnTBAP cotreatment reversed the cisplatin-induced decrease in the DPOAE amplitudes. This indicates that MnTBAP is protective against cisplatin-induced changes in hearing sensitivity and restores the OHC activity.

## Discussion

Cisplatin is widely used to treat numerous types of cancers, which include nasopharyngeal cancer, lung cancer, malignant lymphoma, and other tumors [4]. However, nephrotoxicity, neurotoxicity, and ototoxicity are the side effects of cisplatin, which limit its usage. Amongst these major side effects, ototoxicity manifests as irreversible, progressive neurosensory hearing loss [11]. Hence, it is crucial to develop preventive measures to mitigate cisplatin-induced ototoxicity. The present study indicated that nitrotyrosine levels are elevated in OHCs and SGNs, and cisplatin treatment results in increased hair cell and SGN loss. A strong association between high nitrotyrosine levels and hair cell and SGN loss in the cochlea was observed after cisplatin exposure. More importantly, MnTBAP cotreatment protected hair cells and SGNs from cisplatin-induced death and prevented cisplatin-induced hearing loss.

Oxidative stress plays a crucial role in cisplatin-induced cochlear cell death [11], as NOX3 enzyme is activated and superoxide radicals are produced in the inner ear. Additionally, cisplatin treatment activates the iNOS pathway, resulting in the production of NO in the cochlea [22,23]. The NO reacts with superoxide radicals, resulting in the generation of ONOO-, which increases cochlear nitrative stress, leading to the nitration of many cochlear proteins affecting their normal function. Several studies have reported that cisplatin treatment increases nitrotyrosine and ONOO^-^ levels, which cause apoptosis in the cochlea, leading to hearing loss [48,52]. Consistent with these reports, we observed a significant increase in nitrotyrosine levels in the hair cells and SGNs after cisplatin exposure.

Recent studies have provided potential insights into MnTBAP’s ability to alleviate the toxic side effects of cisplatin chemotherapy. For example, MnTBAP functioned as a mitochondrial ROS scavenger, efficiently decreasing mitochondrial ROS levels while restoring mitochondrial homeostasis in cisplatin-induced acute kidney injury models. It also mitigated oxidative and endoplasmic reticulum (ER) stress in cisplatin-induced nephrotoxicity [53]. In another study, MnTBAP cotreatment preserved cochlear synaptic integrity by reversing the cisplatin-induced decrease in cochlear synaptosomal proteins and attenuating cochlear synaptopathy [54]. Moreover, MnTBAP in its pure form acted as an effective scavenger of ONOO^-^ [45], a critical mediator of the ototoxic effects of cisplatin [48]. In this study, using the same dose used previously to attenuate cisplatin-induced cochlear synaptopathy [54], we observed a strong correlation between cisplatin-induced nitrative stress and hair cell and SGN loss. Effective communication among hair cells, SGNs, and the auditory nerve is essential for normal auditory function [55,56]. However, nitrative stress can promote the degradation of vulnerable nitrated proteins [57,58], including those in the cochlea such as LMO4, a critical target in cisplatin-induced ototoxicity [59]. Therefore, the attenuation of cisplatin-induced cochlear nitrative stress by MnTBAP cotreatment, is consistent with previous observations in organ of Corti cells [60], and along with the preservation of hair cells and SGNs, clearly demonstrates the otoprotective potential of MnTBAP. Although MnTBAP attenuated the cisplatin-induced decrease in LMO4 levels and apoptosis in organ of Corti cells [61], its efficacy in reversing cisplatin-induced nitration of LMO4 was not evaluated, which is a limitation of this study. In follow-up studies, the attenuation of cisplatin-induced nitration of LMO4 by MnTBAP will be studied.

The cisplatin-induced changes in the ABRs and DPOAEs were also consistent with the observed morphological changes in the hair cells and SGNs. Cisplatin treatment elevated hearing thresholds and caused a significant hearing threshold shift on day 8, which is consistent with the threshold shift observed with a cumulative dose of 15 mg/kg in previous reports [48,59]. MnTBAP cotreatment attenuated the cisplatin-induced elevation in hearing thresholds, indicating its ability to preserve auditory signal transmission through the SGNs by protecting them from cisplatin-induced damage. Similarly, DPOAE amplitudes were significantly reduced following cisplatin treatment, whereas MnTBAP cotreatment mitigated this decrease, suggesting a protective effect on OHC function against cisplatin-induced damage. Overall, this study provides both morphological and physiological evidence supporting the therapeutic value of MnTBAP against cisplatin-induced ototoxicity.

## Conclusion

In summary, this study indicated that cotreatment with MnTBAP reversed the cisplatin-induced increase in cochlear nitrative stress, which is implicated in cochlear apoptosis, and protected the hair cells and SGNs from the toxic side effects of cisplatin. Additionally, the results demonstrated that MnTBAP cotreatment attenuated cisplatin-induced hearing loss and reduced the weight loss associated with cisplatin treatment. Collectively, these findings suggest that MnTBAP may serve as an effective interventional compound for mitigating cisplatin-induced ototoxicity. However, further studies are needed to demonstrate whether MnTBAP’s attenuation of cochlear nitrative stress plays a causal role in preventing cisplatin-induced hearing loss. Future research will also evaluate the dose-response, the efficacy of alternative routes of administration, and the effect of MnTBAP treatment on additional cochlear structures and molecular targets.

## Supporting information

S1 Data The raw data for this analysis are available in the S1 Dataset. (XLSX)

## References

[1] Hohnloser JH, Schierl R, Hasford B, Emmerich B. Cisplatin based chemotherapy in testicular cancer patients: long term platinum excretion and clinical effects. Eur J Med Res. 1996;1(11):509–14. 9438151

[2] Helm CW, States JC. Enhancing the efficacy of cisplatin in ovarian cancer treatment - could arsenic have a role. J Ovarian Res. 2009;2:2. doi: 10.1186/1757-2215-2-2 19144189 PMC2636805

[3] Langer T, am Zehnhoff-Dinnesen A, Radtke S, Meitert J, Zolk O. Understanding platinum-induced ototoxicity. Trends Pharmacol Sci. 2013;34(8):458–69. doi: 10.1016/j.tips.2013.05.006 23769626

[4] Dasari S, Tchounwou PB. Cisplatin in cancer therapy: molecular mechanisms of action. Eur J Pharmacol. 2014;740:364–78. doi: 10.1016/j.ejphar.2014.07.025 25058905 PMC4146684

[5] van Zeijl LG, Conijn EA, Rodenburg M, Tange RA, Brocaar MP. Analysis of hearing loss due to cis-diamminedichloroplatinum-II. Arch Otorhinolaryngol. 1984;239(3):255–62. doi: 10.1007/BF00464252 6539589

[6] Knight KRG, Kraemer DF, Neuwelt EA. Ototoxicity in children receiving platinum chemotherapy: underestimating a commonly occurring toxicity that may influence academic and social development. J Clin Oncol. 2005;23(34):8588–96. doi: 10.1200/JCO.2004.00.5355 16314621

[7] Rajput K, Edwards L, Brock P, Abiodun A, Simpkin P, Al-Malky G. Ototoxicity-induced hearing loss and quality of life in survivors of paediatric cancer. Int J Pediatr Otorhinolaryngol. 2020;138:110401. doi: 10.1016/j.ijporl.2020.110401 33152988

[8] Lewis MJ, DuBois SG, Fligor B, Li X, Goorin A, Grier HE. Ototoxicity in children treated for osteosarcoma. Pediatr Blood Cancer. 2009;52(3):387–91. doi: 10.1002/pbc.21875 19061216

[9] Theunissen EAR, Bosma SCJ, Zuur CL, Spijker R, van der Baan S, Dreschler WA, et al. Sensorineural hearing loss in patients with head and neck cancer after chemoradiotherapy and radiotherapy: a systematic review of the literature. Head Neck. 2015;37(2):281–92. doi: 10.1002/hed.23551 24478269

[10] Schmitt NC, Page BR. Chemoradiation-induced hearing loss remains a major concern for head and neck cancer patients. Int J Audiol. 2018;57(sup4):S49–54. doi: 10.1080/14992027.2017.1353710 28728452 PMC6119124

[11] Rybak LP, Whitworth CA, Mukherjea D, Ramkumar V. Mechanisms of cisplatin-induced ototoxicity and prevention. Hear Res. 2007;226(1–2):157–67. doi: 10.1016/j.heares.2006.09.015 17113254

[12] Olgun Y, Aktaş S, Altun Z, Kırkım G, Kızmazoğlu DÇ, Erçetin AP, et al. Analysis of genetic and non genetic risk factors for cisplatin ototoxicity in pediatric patients. Int J Pediatr Otorhinolaryngol. 2016;90:64–9. doi: 10.1016/j.ijporl.2016.09.001 27729156

[13] Anniko M, Sobin A. Cisplatin: evaluation of its ototoxic potential. Am J Otolaryngol. 1986;7(4):276–93. doi: 10.1016/s0196-0709(86)80050-3 3752388

[14] Meech RP, Campbell KC, Hughes LP, Rybak LP. A semiquantitative analysis of the effects of cisplatin on the rat stria vascularis. Hear Res. 1998;124(1–2):44–59. doi: 10.1016/s0378-5955(98)00116-6 9822901

[15] van Ruijven MWM, de Groot JCMJ, Hendriksen F, Smoorenburg GF. Immunohistochemical detection of platinated DNA in the cochlea of cisplatin-treated guinea pigs. Hear Res. 2005;203(1–2):112–21. doi: 10.1016/j.heares.2004.12.007 15855036

[16] Ding D, Allman BL, Salvi R. Review: ototoxic characteristics of platinum antitumor drugs. Anat Rec (Hoboken). 2012;295(11):1851–67. doi: 10.1002/ar.22577 23044998

[17] Steyger PS. Mechanisms of Aminoglycoside- and Cisplatin-Induced Ototoxicity. Am J Audiol. 2021;30(3S):887–900. doi: 10.1044/2021_AJA-21-00006 34415784 PMC9126111

[18] Jamesdaniel S, Ding D, Kermany MH, Davidson BA, Knight PR 3rd, Salvi R, et al. Proteomic analysis of the balance between survival and cell death responses in cisplatin-mediated ototoxicity. J Proteome Res. 2008;7(8):3516–24. doi: 10.1021/pr8002479 18578524 PMC2570323

[19] Park H-J, Kim H-J, Bae G-S, Seo S-W, Kim D-Y, Jung W-S, et al. Selective GSK-3beta inhibitors attenuate the cisplatin-induced cytotoxicity of auditory cells. Hear Res. 2009;257(1–2):53–62. doi: 10.1016/j.heares.2009.08.001 19666099

[20] Schmitt NC, Rubel EW, Nathanson NM. Cisplatin-induced hair cell death requires STAT1 and is attenuated by epigallocatechin gallate. J Neurosci. 2009;29(12):3843–51. doi: 10.1523/JNEUROSCI.5842-08.2009 19321781 PMC2707781

[21] Bánfi B, Malgrange B, Knisz J, Steger K, Dubois-Dauphin M, Krause K-H. NOX3, a superoxide-generating NADPH oxidase of the inner ear. J Biol Chem. 2004;279(44):46065–72. doi: 10.1074/jbc.M403046200 15326186

[22] Li G, Liu W, Frenz D. Cisplatin ototoxicity to the rat inner ear: a role for HMG1 and iNOS. Neurotoxicology. 2006;27(1):22–30. doi: 10.1016/j.neuro.2005.05.010 16125245

[23] Watanabe K, Inai S, Jinnouchi K, Bada S, Hess A, Michel O. Nuclear-factor kappa B (NF-kappa B)-inducible nitric oxide synthase (iNOS/NOS II) pathway damages the stria vascularis in cisplatin-treated mice. Anticancer Research. 2002;22:4081–5. 12553036

[24] Shahab M, Jamesdaniel S. Nitrative Stress and Auditory Dysfunction. Pharmaceuticals (Basel). 2022;15(6):649. doi: 10.3390/ph15060649 35745568 PMC9227425

[25] Novotny-Diermayr V, Lin B, Gu L, Cao X. Modulation of the interleukin-6 receptor subunit glycoprotein 130 complex and its signaling by LMO4 interaction. J Biol Chem. 2005;280(13):12747–57. doi: 10.1074/jbc.M500175200 15677447

[26] Schock SC, Xu J, Duquette PM, Qin Z, Lewandowski AJ, Rai PS, et al. Rescue of neurons from ischemic injury by peroxisome proliferator-activated receptor-gamma requires a novel essential cofactor LMO4. J Neurosci. 2008;28(47):12433–44. doi: 10.1523/JNEUROSCI.2897-08.2008 19020036 PMC2954187

[27] Setogawa T, Shinozaki-Yabana S, Masuda T, Matsuura K, Akiyama T. The tumor suppressor LKB1 induces p21 expression in collaboration with LMO4, GATA-6, and Ldb1. Biochem Biophys Res Commun. 2006;343(4):1186–90. doi: 10.1016/j.bbrc.2006.03.077 16580634

[28] Wang N, Lin KK, Lu Z, Lam KS, Newton R, Xu X, et al. The LIM-only factor LMO4 regulates expression of the BMP7 gene through an HDAC2-dependent mechanism, and controls cell proliferation and apoptosis of mammary epithelial cells. Oncogene. 2007;26(44):6431–41. doi: 10.1038/sj.onc.1210465 17452977

[29] Deng M, Luo X, Pan L, Yang H, Xie X, Liang G, et al. LMO4 functions as a negative regulator of sensory organ formation in the mammalian cochlea. J Neurosci. 2014;34(30):10072–7. doi: 10.1523/JNEUROSCI.0352-14.2014 25057208 PMC4107398

[30] Ramkumar V, Mukherjea D, Dhukhwa A, Rybak LP. Oxidative Stress and Inflammation Caused by Cisplatin Ototoxicity. Antioxidants (Basel). 2021;10(12):1919. doi: 10.3390/antiox10121919 34943021 PMC8750101

[31] Yu D, Gu J, Chen Y, Kang W, Wang X, Wu H. Current Strategies to Combat Cisplatin-Induced Ototoxicity. Front Pharmacol. 2020;11:999. doi: 10.3389/fphar.2020.00999 32719605 PMC7350523

[32] Bhatta P, Dhukhwa A, Sheehan K, Al Aameri RFH, Borse V, Ghosh S, et al. Capsaicin Protects Against Cisplatin Ototoxicity by Changing the STAT3/STAT1 Ratio and Activating Cannabinoid (CB2) Receptors in the Cochlea. Sci Rep. 2019;9(1):4131. doi: 10.1038/s41598-019-40425-9 30858408 PMC6411993

[33] Kim H-J, Oh G-S, Lee J-H, Lyu A-R, Ji H-M, Lee S-H, et al. Cisplatin ototoxicity involves cytokines and STAT6 signaling network. Cell Res. 2011;21(6):944–56. doi: 10.1038/cr.2011.27 21321603 PMC3203698

[34] Plane J, Cabral TDD, Knoll RM, Conrado JEP, Vendramini BDV, Jung DH. N-acetylcysteine for the Prevention of Cisplatin-Induced Hearing Loss: A Systematic Review and Meta-analysis. Otolaryngol Head Neck Surg. 2025;173(2):345–59. doi: 10.1002/ohn.1272 40323204 PMC13095053

[35] Meijer AJM, Diepstraten FA, Ansari M, Bouffet E, Bleyer A, Fresneau B, et al. Use of Sodium Thiosulfate as an Otoprotectant in Patients With Cancer Treated With Platinum Compounds: A Review of the Literature. J Clin Oncol. 2024;42(18):2219–32. doi: 10.1200/JCO.23.02353 38648563 PMC11191063

[36] Orgel E, Knight KR, Chi Y-Y, Malvar J, Rushing T, Mena V, et al. Intravenous N-Acetylcysteine to Prevent Cisplatin-Induced Hearing Loss in Children: A Nonrandomized Controlled Phase I Trial. Clin Cancer Res. 2023;29(13):2410–8. doi: 10.1158/1078-0432.CCR-23-0252 37134194 PMC10330342

[37] Rolland V, Meyer F, Guitton MJ, Bussières R, Philippon D, Bairati I, et al. A randomized controlled trial to test the efficacy of trans-tympanic injections of a sodium thiosulfate gel to prevent cisplatin-induced ototoxicity in patients with head and neck cancer. J Otolaryngol Head Neck Surg. 2019;48(1):4. doi: 10.1186/s40463-019-0327-x 30651130 PMC6335693

[38] Sarafraz Z, Ahmadi A, Daneshi A. Transtympanic Injections of N-acetylcysteine and Dexamethasone for Prevention of Cisplatin-Induced Ototoxicity: Double Blind Randomized Clinical Trial. Int Tinnitus J. 2018;22(1):40–5. doi: 10.5935/0946-5448.20180007 29993216

[39] Viale M, Zhang JG, Pastrone I, Mariggiò MA, Esposito M, Lindup WE. Cisplatin combined with tiopronin or sodium thiosulfate: cytotoxicity in vitro and antitumor activity in vivo. Anticancer Drugs. 1999;10(4):419–28. doi: 10.1097/00001813-199904000-00011 10378678

[40] Wu YJ, Muldoon LL, Neuwelt EA. The chemoprotective agent N-acetylcysteine blocks cisplatin-induced apoptosis through caspase signaling pathway. J Pharmacol Exp Ther. 2005;312(2):424–31. doi: 10.1124/jpet.104.075119 15496615

[41] Brock PR, Maibach R, Childs M, Rajput K, Roebuck D, Sullivan MJ, et al. Sodium Thiosulfate for Protection from Cisplatin-Induced Hearing Loss. N Engl J Med. 2018;378(25):2376–85. doi: 10.1056/NEJMoa1801109 29924955 PMC6117111

[42] El Kechai N, Agnely F, Mamelle E, Nguyen Y, Ferrary E, Bochot A. Recent advances in local drug delivery to the inner ear. Int J Pharm. 2015;494(1):83–101. doi: 10.1016/j.ijpharm.2015.08.015 26260230

[43] Müller U, Barr-Gillespie PG. New treatment options for hearing loss. Nat Rev Drug Discov. 2015;14(5):346–65. doi: 10.1038/nrd4533 25792261

[44] van As JW, van den Berg H, van Dalen EC. Medical interventions for the prevention of platinum-induced hearing loss in children with cancer. Cochrane Database Syst Rev. 2019;5(5):CD009219. doi: 10.1002/14651858.CD009219.pub5 31063591 PMC6504134

[45] Batinić-Haberle I, Cuzzocrea S, Rebouças JS, Ferrer-Sueta G, Mazzon E, Di Paola R, et al. Pure MnTBAP selectively scavenges peroxynitrite over superoxide: comparison of pure and commercial MnTBAP samples to MnTE-2-PyP in two models of oxidative stress injury, an SOD-specific Escherichia coli model and carrageenan-induced pleurisy. Free Radic Biol Med. 2009;46(2):192–201. doi: 10.1016/j.freeradbiomed.2008.09.042 19007878 PMC2742324

[46] Pan H, Shen K, Wang X, Meng H, Wang C, Jin B. Protective effect of metalloporphyrins against cisplatin-induced kidney injury in mice. PLoS One. 2014;9:e86057. doi: 10.1371/journal.pone.0086057 PMC3891880 24454954

[47] Klein M, Koedel U, Pfister H-W, Kastenbauer S. Meningitis-associated hearing loss: protection by adjunctive antioxidant therapy. Ann Neurol. 2003;54(4):451–8. doi: 10.1002/ana.10684 14520656

[48] Jamesdaniel S, Rathinam R, Neumann WL. Targeting nitrative stress for attenuating cisplatin-induced downregulation of cochlear LIM domain only 4 and ototoxicity. Redox Biol. 2016;10:257–65. doi: 10.1016/j.redox.2016.10.016 27821327 PMC5099269

[49] Fernandez K, Wafa T, Fitzgerald TS, Cunningham LL. An optimized, clinically relevant mouse model of cisplatin-induced ototoxicity. Hear Res. 2019;375:66–74. doi: 10.1016/j.heares.2019.02.006 30827780 PMC6416072

[50] Han W, Shi X, Nuttall A. Noise-induced nitrotyrosine increase and outer hair cell death in guinea pig cochlea. Chin Med J (Engl). 2013;126(15):2923–7. 23924469 PMC3947561

[51] Hu Z, Komal F, Singh A, Deng M. Generation of a Spiral Ganglion Neuron Degeneration Mouse Model. Front Cell Dev Biol. 2021;9:761847. doi: 10.3389/fcell.2021.761847 34778272 PMC8578993

[52] Jamesdaniel S, Coling D, Hinduja S, Ding D, Li J, Cassidy L, et al. Cisplatin-induced ototoxicity is mediated by nitroxidative modification of cochlear proteins characterized by nitration of Lmo4. J Biol Chem. 2012;287(22):18674–86. doi: 10.1074/jbc.M111.297960 22493493 PMC3365719

[53] Li Y, Jiang Y, Zhou W, Wu Y, Zhang S, Ding G, et al. Maintaining homeostasis of mitochondria and endoplasmic reticulum with NSC228155 alleviates cisplatin-induced acute kidney injury. Free Radic Biol Med. 2022;181:270–87. doi: 10.1016/j.freeradbiomed.2022.02.003 35134531

[54] Shahab M, Rosati R, Stemmer PM, Dombkowski A, Jamesdaniel S. Quantitative profiling of cochlear synaptosomal proteins in cisplatin-induced synaptic dysfunction. Hear Res. 2024;447:109022. doi: 10.1016/j.heares.2024.109022 38705005 PMC11116033

[55] Raphael Y, Altschuler RA. Structure and innervation of the cochlea. Brain Res Bull. 2003;60(5–6):397–422. doi: 10.1016/s0361-9230(03)00047-9 12787864

[56] Monzack EL, Cunningham LL. Lead roles for supporting actors: critical functions of inner ear supporting cells. Hear Res. 2013;303:20–9. doi: 10.1016/j.heares.2013.01.008 23347917 PMC3648608

[57] Radi R. Protein tyrosine nitration: biochemical mechanisms and structural basis of functional effects. Acc Chem Res. 2013;46(2):550–9. doi: 10.1021/ar300234c 23157446 PMC3577981

[58] Souza JM, Peluffo G, Radi R. Protein tyrosine nitration--functional alteration or just a biomarker? Free Radic Biol Med. 2008;45(4):357–66. doi: 10.1016/j.freeradbiomed.2008.04.010 18460345

[59] Rosati R, Shahab M, Ramkumar V, Jamesdaniel S. Lmo4 Deficiency Enhances Susceptibility to Cisplatin-Induced Cochlear Apoptosis and Hearing Loss. Mol Neurobiol. 2021;58(5):2019–29. doi: 10.1007/s12035-020-02226-4 33411315 PMC8026651

[60] Mehmood S, Bhatia P, Doyon-Reale N, Jamesdaniel S. Differential effect of targeting cisplatin-induced nitrative stress using MnTBAP in auditory and cancer cells. Curr Res Toxicol. 2025;8:100241. doi: 10.1016/j.crtox.2025.100241 40496981 PMC12148825

[61] Shahab M, Rosati R, Bhatia P, Doyon-Reale N, Jamesdaniel S. MnTBAP, a peroxynitrite scavenger, attenuates cisplatin-induced apoptosis and cytotoxicity in organ of Corti cells. Toxicol Rep. 2025;14:101967. doi: 10.1016/j.toxrep.2025.101967 40046635 PMC11880612

