# Supplementary material for: Otoprotective effect of MnTBAP in cisplatin-induced hearing loss
Source: PLoS One. 2026 Sep 21;21(9):e0351911. doi: 10.1371/journal.pone.0351911 (PMC13592571; doi:10.1371/journal.pone.0351911)
Supplement: Supplementary file 1 [file pone.0351911.s003.docx]

**Response to Editor Comments :**
The reviewers consider the manuscript potentially valuable and agree that the study provides evidence supporting an otoprotective effect of MnTBAP against cisplatin-induced cochlear injury. However, both reviewers identified several issues that should be addressed before the manuscript can be considered further.

*In particular, the authors should clarify the experimental and statistical methodology, including the sample sizes for individual auditory outcome measures, the handling of measurements from the two ears, the treatment of repeated auditory measurements, and whether sex was considered as a biological variable. The rationale for the selected cisplatin and MnTBAP doses should also be provided.*

We have added more information about the experimental and statistical methodology, particularly about sample sizes, ears from which auditory measurements were recorded, sex as a biological variable, and the rationale for cisplatin and MnTBAP doses.

*The interpretation of the nitrotyrosine findings should be revised to distinguish clearly between association and causal mechanistic evidence. The current data support an association between reduced nitrative stress and protection against cisplatin-induced cochlear injury, but do not by themselves establish a direct causal role of nitrotyrosine in hair-cell or spiral ganglion neuron apoptosis. The authors should therefore moderate mechanistic claims and appropriately acknowledge this limitation.*

We have moderated the interpretation about nitrotyrosine to more accurately reflect the association.

*The Data Availability Statement must also be revised to comply with PLOS ONE requirements by making the underlying data fully accessible through an appropriate public repository or Supporting Information. The authors should additionally address the remaining issues concerning figure quality and statistical annotations, terminology, language clarity, and reference formatting.*

We have provided the raw data as supplementary material. We have also enhanced figure quality, provided statistical annotations, clarified terminology, and formatted the references.

*Additional experiments suggested by the reviewers, such as alternative routes of MnTBAP administration, dose-response studies, or additional cochlear structural markers, may be informative but are not necessarily required to address the central question of the present study. If such experiments are not performed, the relevant limitations and directions for future research should be clearly acknowledged.*

We have mentioned that the additional studies will be conducted in follow-up or future studies.

*Please provide a detailed point-by-point response to both reviewers and indicate all changes made in the revised manuscript.*

We have provided a point-by-point response to the reviewers' comments.

**Response to Reviewers' comments:**

*Reviewer #1: In this study, CBA/J mice were used as animal models to conduct in vivo experiments investigating the protective effect of MnTBAP against cisplatin-induced ototoxicity. Centered on the nitrosative stress pathway, we combined cochlear morphological and auditory functional indicators to verify that co-administration of MnTBAP alleviates cisplatin-triggered cochlear nitrosative stress, reduces the loss of hair cells and spiral ganglion neurons, mitigates hearing threshold shifts, and reverses cisplatin-induced weight loss in mice. Correlation analysis further confirmed a significant positive correlation between nitrotyrosine accumulation and cochlear cellular damage. This research has certain scientific significance, yet several limitations remain to be addressed.*

Major problems:
*1. The study only quantified the total fluorescence intensity of nitrotyrosine without targeted mechanistic verification, which fails to demonstrate that nitrotyrosine accumulation directly mediates apoptosis of hair cells and spiral ganglion neurons.*

We have cited additional references in which we demonstrated cisplatin-induced nitrative stress and apoptosis in organ of Corti cells to support the potential association between nitrative stress and cell death in the cochlea.
*2. Morphological detection is insufficient: only Myosin-VIIa was applied to count inner and outer hair cells, and Phalloidin was used to label SGNs. The integrity of outer hair cell stereocilia and damage to cochlear ribbon synapses were not evaluated. Additional relevant biomarkers are required to comprehensively validate the protective efficacy of MnTBAP against cisplatin-induced hearing loss.*

We have noted this as a limitation of this study and indicated that future studies will examine additional cochlear structures and molecular targets.
*3. Safety evaluation is incomplete. Monitoring body weight and hearing function alone cannot fully assess the in vivo safety of MnTBAP. Serum biochemical indicators reflecting liver and renal function should be supplemented.*

We have clarified that although evaluation of liver and renal function is required to thoroughly investigate the toxicity profile of the compound, body weight monitoring can provide some insights about systemic toxicity.
*4. Only intraperitoneal injection was adopted in this work and proved effective. Further investigations on round window administration are recommended to compare the therapeutic performance of MnTBAP in different delivery routes.*

We have noted that alternative routes of administration will be used in our future studies to further evaluate the therapeutic efficacy of MnTBAP.

Minor problems:
*1. The rationale for selecting the 10 mg/kg dosage of MnTBAP was not explained. Dose-gradient groups are absent, so it remains unclear whether other concentrations exert protective effects against cisplatin-induced hearing impairment.*

We have provided the rationale for the MnTBAP dose and cited our previous research article to support it.
*2. The resolution of provided figures is inadequate; high-quality original images should be supplied.*

We have enhanced the resolution of the figures. The quality of the images in the Tiff files is much better than how it appears in the PDF document.
*3. The manuscript contains numerous lengthy complex sentences with repetitive descriptions. It is suggested to split long sentences, eliminate redundant expressions and streamline the whole text.*

As suggested, we have shortened the lengthy sentences and removed repetitions, and revised the whole text.
*4. Incorporating the relevant references listed below (PMID: 38512388, PMID: 40387257) would help further strengthen the scientific rigor of this manuscript.*

We have cited additional relevant references to strengthen the manuscript. We refrained from adding the suggested references as these articles were about nanoparticles and radionuclide-labeled antibodies.

*Reviewer #2: 1. Terminology and consistency require careful revision
There appears to be an incorrect use of terminology in several sections of the manuscript. The term “nitrofurylone” appears inconsistent with the experimental description and should be carefully checked throughout the manuscript. Based on the context, the authors likely intended to refer to nitrotyrosine, which was the marker evaluated for nitrative stress. This terminology error should be corrected throughout the manuscript to avoid confusion.
The authors should also ensure consistent use of oxidative/nitrative stress-related terminology, including nitrotyrosine and peroxynitrite (ONOO−).*

We have double-checked the manuscript to ensure that the term “nitrotyrosine” is not misspelled. We have described ONOO− as peroxynitrite at the first instance, and thereafter used the abbreviation consistently throughout the manuscript.

*2. Experimental details require further clarification
Several methodological details should be expanded:
The authors should provide additional information regarding MnTBAP, including batch information and purity if available. This is important because previous studies have indicated that MnTBAP activity may vary depending on compound purity.
The rationale for selecting the cisplatin and MnTBAP doses should be clarified, preferably with supporting references or preliminary data.
Although the authors indicate that male and female mice were balanced among groups, it remains unclear whether sex-related effects were evaluated. The authors should clarify whether sex was considered as a biological variable or statistical factor.*

We have provided additional information regarding MnTBAP’s purity, Lot number, and included the rationale for cisplatin and MnTBAP doses. We have also provided the number of males and females used in the figure legends and clarified that we did not consider sex as a biological variable because of the low sample size in each sex.

*3. Statistical analysis and data presentation need improvement
Several aspects of statistical reporting require clarification:
The exact sample size for each experiment should be clearly indicated. For ABR and DPOAE analyses, the manuscript should specify the number of animals included at each frequency and whether measurements were obtained from one ear or both ears.
If both ears were analyzed, the authors should clarify whether ears were treated as independent observations or averaged per animal.
The statistical approach for repeated auditory measurements should be described more clearly, including whether repeated measures were considered in the analysis.
Correlation analyses shown in Figure 5 are based on a relatively small sample size (n=5). The authors should acknowledge this limitation and interpret these findings cautiously.
The statistical symbols in several figures are also inconsistent or excessive. The authors should revise figure annotations using standard statistical notation (p<0.05, p<0.01, p<0.001, ****p<0.0001) and clearly define all symbols in figure legends.*

We have improved the statistical analysis and data presentation as suggested. We included the sample size in the figure legends and indicated the ear from which the data were collected. We have also included the limitations in the data analysis for those that had a small sample size and revised the figure annotations correctly.

*4. Mechanistic conclusions should be interpreted more cautiously
The authors propose that MnTBAP protects against cisplatin ototoxicity through reduction of nitrative stress. Although increased nitrotyrosine levels correlate with cochlear damage, correlation does not establish a direct causal mechanism.
The manuscript would benefit from either additional mechanistic experiments (e.g., apoptosis-related markers, direct assessment of peroxynitrite-related pathways) or a more cautious interpretation of the findings. The conclusion should emphasize that MnTBAP is associated with reduced nitrative stress and cochlear protection rather than definitively proving the underlying mechanism.
The relationship between MnTBAP treatment and previously reported targets such as LMO4 nitration should also be clarified. If MnTBAP has not been directly demonstrated to regulate LMO4 modification, this should be stated as a potential future research direction rather than an established mechanism.*

We have moderated the statements regarding the mechanistic interpretations and emphasized that the data indicate a correlation rather than causation. We have also noted that in follow-up studies we will investigate the effect of MnTBAP on Cisplatin-induced nitration of LMO4.

*5. Data availability statement does not comply with PLOS ONE requirements
The current statement indicates that datasets will be “made available on request.” However, PLOS ONE requires underlying data to be publicly available without restriction.
The authors should revise the Data Availability Statement by depositing the raw data in an appropriate public repository (e.g., Figshare, Dryad, or Zenodo) and providing accession information or DOI. Alternatively, the authors should provide the relevant datasets as supplementary materials.*

As suggested, we have provided the datasets as supplementary material.

Minor Comments
*1.The abstract would benefit from clearer separation of Methods and Results sections and improved readability.*

We have revised the abstract to enhance clarity.
*2.Figure legends should clearly define all symbols, abbreviations, and statistical comparisons, particularly the meaning of “#” symbols.*

We have revised all figure legends to define abbreviations and symbols.
*3.The manuscript should be carefully edited for language clarity. Several sentences are lengthy and could be simplified for better readability.*

We have edited the manuscript carefully and replaced lengthy sentences with simple sentences.
*4.The authors should discuss whether the observed reduction in cisplatin-induced body weight loss by MnTBAP reflects reduced systemic toxicity, improved general condition, or other factors.*

We have included a discussion of potential reasons for changes in the animals' body weights.
*5.The ethical statement should clarify whether animal procedures followed relevant guidelines such as AVMA recommendations for euthanasia.*

We have added the clarification.
*6.Reference formatting should be carefully checked and standardized according to PLOS ONE requirements.*

References have been checked.

The changes made in response to the reviewer’s comments have improved and strengthened the manuscript. We request to consider the revised manuscript for publication.
