# Supplementary material for: Otoprotective effect of MnTBAP in cisplatin-induced hearing loss
Source: PLoS One. 2026 Sep 21;21(9):e0351911. doi: 10.1371/journal.pone.0351911 (PMC13592571; doi:10.1371/journal.pone.0351911)
Supplement: Supplementary file 2 [file pone.0351911.s004.docx]

**Response to Journal and Editor Comments**

Journal Requirements:

*If the reviewer comments include a recommendation to cite specific previously published works, please review and evaluate these publications to determine whether they are relevant and should be cited. There is no requirement to cite these works unless the editor has indicated otherwise.*

We reviewed and refrained from adding two references suggested by the reviewer (PMID: 38512388, PMID: 40387257), as these articles were about nanoparticles and radionuclide-labeled antibodies.

*Please review your reference list to ensure that it is complete and correct. If you have cited papers that have been retracted, please include the rationale for doing so in the manuscript text, or remove these references and replace them with relevant current references. Any changes to the reference list should be mentioned in the rebuttal letter that accompanies your revised manuscript. If you need to cite a retracted article, indicate the article’s retracted status in the References list and also include a citation and full reference for the retraction notice.*

We reviewed the reference list and have corrected the errors. None of the cited papers have been retracted.

Additional Editor Comments:

*The revised manuscript has addressed the major scientific and methodological concerns raised during peer review. The additional methodological details, clarification of the experimental procedures, expanded ethical statement, revised Data Availability Statement, and more cautious discussion have substantially strengthened the manuscript.
Before acceptance, the authors should address a few remaining editorial issues, including clarification of the auditory data presentation (left ear vs bilateral measurements where appropriate) and slightly moderating the wording of the conclusions regarding the therapeutic potential of MnTBAP.*

We have clarified in the methods section under statistical analysis that we used the data generated from the left ear and did not include bilateral measurements in the analysis. We have also moderated the wording of the conclusions.

We request to consider the revised manuscript for publication.
