# Supplementary material for: Otoprotective effect of MnTBAP in cisplatin-induced hearing loss
Source: PLoS One. 2026 Sep 21;21(9):e0351911. doi: 10.1371/journal.pone.0351911 (PMC13592571; doi:10.1371/journal.pone.0351911)
Supplement: Peer review history

# Peer review: Decision Letter 0

**DOI:** 10.1371/journal.pone.0351911.r001

**Authors:** Pavle Randjelovic

12 Aug 2026

Dear Dr. Jamesdaniel,

Thank you for submitting your manuscript to PLOS ONE. After careful consideration, we feel that it has merit but does not fully meet PLOS ONE’s publication criteria as it currently stands. Therefore, we invite you to submit a revised version of the manuscript that addresses the points raised during the review process.

Please submit your revised manuscript by Oct 11 2026 11:59PM. If you will need more time than this to complete your revisions, please reply to this message or contact the journal office at plosone@plos.org. When you're ready to submit your revision, log on to https://www.editorialmanager.com/pone/ and select the 'Submissions Needing Revision' folder to locate your manuscript file.

- A letter that responds to each point raised by the academic editor and reviewer(s). You should upload this letter as a separate file labeled 'Response to Reviewers'.
- A marked-up copy of your manuscript that highlights changes made to the original version. You should upload this as a separate file labeled 'Revised Manuscript with Track Changes'.
- An unmarked version of your revised paper without tracked changes. You should upload this as a separate file labeled 'Manuscript'.

If you would like to make changes to your financial disclosure, please include your updated statement in your cover letter. Guidelines for resubmitting your figure files are available below the reviewer comments at the end of this letter.

If applicable, we recommend that you deposit your laboratory protocols in protocols.io to enhance the reproducibility of your results. Protocols.io assigns your protocol its own identifier (DOI) so that it can be cited independently in the future. For instructions see: https://journals.plos.org/plosone/s/submission-guidelines#loc-laboratory-protocols. Additionally, PLOS ONE offers an option for publishing peer-reviewed Lab Protocol articles, which describe protocols hosted on protocols.io. Read more information on sharing protocols at https://plos.org/protocols?utm_medium=editorial-email&utm_source=authorletters&utm_campaign=protocols.

As the corresponding author, your ORCID iD is verified in the submission system and will appear in the published article. PLOS supports the use of ORCID, and we encourage all coauthors to register for an ORCID iD and use it as well. Please encourage your coauthors to verify their ORCID iD within the submission system before final acceptance, as unverified ORCID iDs will not appear in the published article. *Only*  the individual author can complete the verification step; PLOS staff *cannot*  verify ORCID iDs on behalf of authors.

We look forward to receiving your revised manuscript.

Kind regards,

Pavle Randjelovic, Ph.D.

Academic Editor

PLOS One

Journal Requirements:

When submitting your revision, we need you to address these additional requirements.

1. Please ensure that your manuscript meets PLOS ONE's style requirements, including those for file naming. The PLOS ONE style templates can be found at

https://journals.plos.org/plosone/s/file?id=wjVg/PLOSOne_formatting_sample_main_body.pdf and https://journals.plos.org/plosone/s/file?id=ba62/PLOSOne_formatting_sample_title_authors_affiliations.pdf

2. In the online submission form, you indicated that the datasets used and analyzed in the current study will be made available on request.

All PLOS journals now require all data underlying the findings described in their manuscript to be freely available to other researchers, either 1. In a public repository, 2. Within the manuscript itself, or 3. Uploaded as supplementary information.

This policy applies to all data except where public deposition would breach compliance with the protocol approved by your research ethics board. If your data cannot be made publicly available for ethical or legal reasons (e.g., public availability would compromise patient privacy), please explain your reasons on resubmission and your exemption request will be escalated for approval.

3. If the reviewer comments include a recommendation to cite specific previously published works, please review and evaluate these publications to determine whether they are relevant and should be cited. There is no requirement to cite these works unless the editor has indicated otherwise.

Additional Editor Comments :

The reviewers consider the manuscript potentially valuable and agree that the study provides evidence supporting an otoprotective effect of MnTBAP against cisplatin-induced cochlear injury. However, both reviewers identified several issues that should be addressed before the manuscript can be considered further.

In particular, the authors should clarify the experimental and statistical methodology, including the sample sizes for individual auditory outcome measures, the handling of measurements from the two ears, the treatment of repeated auditory measurements, and whether sex was considered as a biological variable. The rationale for the selected cisplatin and MnTBAP doses should also be provided.

The interpretation of the nitrotyrosine findings should be revised to distinguish clearly between association and causal mechanistic evidence. The current data support an association between reduced nitrative stress and protection against cisplatin-induced cochlear injury, but do not by themselves establish a direct causal role of nitrotyrosine in hair-cell or spiral ganglion neuron apoptosis. The authors should therefore moderate mechanistic claims and appropriately acknowledge this limitation.

The Data Availability Statement must also be revised to comply with PLOS ONE requirements by making the underlying data fully accessible through an appropriate public repository or Supporting Information. The authors should additionally address the remaining issues concerning figure quality and statistical annotations, terminology, language clarity, and reference formatting.

Additional experiments suggested by the reviewers, such as alternative routes of MnTBAP administration, dose-response studies, or additional cochlear structural markers, may be informative but are not necessarily required to address the central question of the present study. If such experiments are not performed, the relevant limitations and directions for future research should be clearly acknowledged.

Please provide a detailed point-by-point response to both reviewers and indicate all changes made in the revised manuscript.

[Note: HTML markup is below. Please do not edit.]

Reviewers' comments:

Reviewer's Responses to Questions

**Comments to the Author**

1. Is the manuscript technically sound, and do the data support the conclusions?

Reviewer #1: Yes

Reviewer #2: Yes

**********

2. Has the statistical analysis been performed appropriately and rigorously?

Reviewer #1: Yes

Reviewer #2: Yes

**********

3. Have the authors made all data underlying the findings in their manuscript fully available?

The PLOS Data policy

Reviewer #1: Yes

Reviewer #2: Yes

**********

4. Is the manuscript presented in an intelligible fashion and written in standard English?

Reviewer #1: Yes

Reviewer #2: Yes

**********

Reviewer #1: In this study, CBA/J mice were used as animal models to conduct in vivo experiments investigating the protective effect of MnTBAP against cisplatin-induced ototoxicity. Centered on the nitrosative stress pathway, we combined cochlear morphological and auditory functional indicators to verify that co-administration of MnTBAP alleviates cisplatin-triggered cochlear nitrosative stress, reduces the loss of hair cells and spiral ganglion neurons, mitigates hearing threshold shifts, and reverses cisplatin-induced weight loss in mice. Correlation analysis further confirmed a significant positive correlation between nitrotyrosine accumulation and cochlear cellular damage. This research has certain scientific significance, yet several limitations remain to be addressed.

Major problems:

1. The study only quantified the total fluorescence intensity of nitrotyrosine without targeted mechanistic verification, which fails to demonstrate that nitrotyrosine accumulation directly mediates apoptosis of hair cells and spiral ganglion neurons.

2. Morphological detection is insufficient: only Myosin-VIIa was applied to count inner and outer hair cells, and Phalloidin was used to label SGNs. The integrity of outer hair cell stereocilia and damage to cochlear ribbon synapses were not evaluated. Additional relevant biomarkers are required to comprehensively validate the protective efficacy of MnTBAP against cisplatin-induced hearing loss.

3. Safety evaluation is incomplete. Monitoring body weight and hearing function alone cannot fully assess the in vivo safety of MnTBAP. Serum biochemical indicators reflecting liver and renal function should be supplemented.

4. Only intraperitoneal injection was adopted in this work and proved effective. Further investigations on round window administration are recommended to compare the therapeutic performance of MnTBAP in different delivery routes.

Minor problems:

1. The rationale for selecting the 10 mg/kg dosage of MnTBAP was not explained. Dose-gradient groups are absent, so it remains unclear whether other concentrations exert protective effects against cisplatin-induced hearing impairment.

2. The resolution of provided figures is inadequate; high-quality original images should be supplied.

3. The manuscript contains numerous lengthy complex sentences with repetitive descriptions. It is suggested to split long sentences, eliminate redundant expressions and streamline the whole text.

4. Incorporating the relevant references listed below (PMID: 38512388, PMID: 40387257) would help further strengthen the scientific rigor of this manuscript.

Reviewer #2: 1. Terminology and consistency require careful revision

There appears to be an incorrect use of terminology in several sections of the manuscript. The term “nitrofurylone” appears inconsistent with the experimental description and should be carefully checked throughout the manuscript. Based on the context, the authors likely intended to refer to nitrotyrosine, which was the marker evaluated for nitrative stress. This terminology error should be corrected throughout the manuscript to avoid confusion.

The authors should also ensure consistent use of oxidative/nitrative stress-related terminology, including nitrotyrosine and peroxynitrite (ONOO−).

2. Experimental details require further clarification

Several methodological details should be expanded:

The authors should provide additional information regarding MnTBAP, including batch information and purity if available. This is important because previous studies have indicated that MnTBAP activity may vary depending on compound purity.

The rationale for selecting the cisplatin and MnTBAP doses should be clarified, preferably with supporting references or preliminary data.

Although the authors indicate that male and female mice were balanced among groups, it remains unclear whether sex-related effects were evaluated. The authors should clarify whether sex was considered as a biological variable or statistical factor.

3. Statistical analysis and data presentation need improvement

Several aspects of statistical reporting require clarification:

The exact sample size for each experiment should be clearly indicated. For ABR and DPOAE analyses, the manuscript should specify the number of animals included at each frequency and whether measurements were obtained from one ear or both ears.

If both ears were analyzed, the authors should clarify whether ears were treated as independent observations or averaged per animal.

The statistical approach for repeated auditory measurements should be described more clearly, including whether repeated measures were considered in the analysis.

Correlation analyses shown in Figure 5 are based on a relatively small sample size (n=5). The authors should acknowledge this limitation and interpret these findings cautiously.

The statistical symbols in several figures are also inconsistent or excessive. The authors should revise figure annotations using standard statistical notation (p<0.05, p<0.01, p<0.001, ****p<0.0001) and clearly define all symbols in figure legends.

4. Mechanistic conclusions should be interpreted more cautiously

The authors propose that MnTBAP protects against cisplatin ototoxicity through reduction of nitrative stress. Although increased nitrotyrosine levels correlate with cochlear damage, correlation does not establish a direct causal mechanism.

The manuscript would benefit from either additional mechanistic experiments (e.g., apoptosis-related markers, direct assessment of peroxynitrite-related pathways) or a more cautious interpretation of the findings. The conclusion should emphasize that MnTBAP is associated with reduced nitrative stress and cochlear protection rather than definitively proving the underlying mechanism.

The relationship between MnTBAP treatment and previously reported targets such as LMO4 nitration should also be clarified. If MnTBAP has not been directly demonstrated to regulate LMO4 modification, this should be stated as a potential future research direction rather than an established mechanism.

5. Data availability statement does not comply with PLOS ONE requirements

The current statement indicates that datasets will be “made available on request.” However, PLOS ONE requires underlying data to be publicly available without restriction.

The authors should revise the Data Availability Statement by depositing the raw data in an appropriate public repository (e.g., Figshare, Dryad, or Zenodo) and providing accession information or DOI. Alternatively, the authors should provide the relevant datasets as supplementary materials.

Minor Comments

1.The abstract would benefit from clearer separation of Methods and Results sections and improved readability.

2.Figure legends should clearly define all symbols, abbreviations, and statistical comparisons, particularly the meaning of “#” symbols.

3.The manuscript should be carefully edited for language clarity. Several sentences are lengthy and could be simplified for better readability.

4.The authors should discuss whether the observed reduction in cisplatin-induced body weight loss by MnTBAP reflects reduced systemic toxicity, improved general condition, or other factors.

5.The ethical statement should clarify whether animal procedures followed relevant guidelines such as AVMA recommendations for euthanasia.

6.Reference formatting should be carefully checked and standardized according to PLOS ONE requirements.

**********

what does this mean?). If published, this will include your full peer review and any attached files.

If you choose “no”, your identity will remain anonymous but your review may still be made public.

**Do you want your identity to be public for this peer review?**  For information about this choice, including consent withdrawal, please see our Privacy Policy

Reviewer #1: No

Reviewer #2: No

**********

[NOTE: If reviewer comments were submitted as an attachment file, they will be attached to this email and accessible via the submission site. Please log into your account, locate the manuscript record, and check for the action link "View Attachments". If this link does not appear, there are no attachment files.]

To ensure your figures meet our technical requirements, please review our figure guidelines: https://journals.plos.org/plosone/s/figures

You may also use PLOS’s free figure tool, NAAS, to help you prepare publication quality figures: https://journals.plos.org/plosone/s/figures#loc-tools-for-figure-preparation.

NAAS will assess whether your figures meet our technical requirements by comparing each figure against our figure specifications.

# Author comment: Author response to Decision Letter 1

**DOI:** 10.1371/journal.pone.0351911.r002

18 Aug 2026

We have carefully addressed all reviewers' comments and have provided a point-by-point response.

Attachment Submitted filename: Response to Reviewers Comments.docx

# Editor report: Decision Letter 1

**DOI:** 10.1371/journal.pone.0351911.r003

**Authors:** Pavle Randjelovic

22 Aug 2026

Dear Dr. Jamesdaniel,

Thank you for submitting your manuscript to PLOS One. After careful consideration, we feel that it has merit but does not fully meet PLOS One’s publication criteria as it currently stands. Therefore, we invite you to submit a revised version of the manuscript that addresses the points raised during the review process.

Please submit your revised manuscript by Sep 21 2026 11:59PM. If you will need more time than this to complete your revisions, please reply to this message or contact the journal office at plosone@plos.org. When you're ready to submit your revision, log on to https://www.editorialmanager.com/pone/ and select the 'Submissions Needing Revision' folder to locate your manuscript file.

- A letter that responds to each point raised by the academic editor and reviewer(s). You should upload this letter as a separate file labeled 'Response to Reviewers'.
- A marked-up copy of your manuscript that highlights changes made to the original version. You should upload this as a separate file labeled 'Revised Manuscript with Track Changes'.
- An unmarked version of your revised paper without tracked changes. You should upload this as a separate file labeled 'Manuscript'.

If you would like to make changes to your financial disclosure, please include your updated statement in your cover letter. Guidelines for resubmitting your figure files are available below the reviewer comments at the end of this letter.

If applicable, we recommend that you deposit your laboratory protocols in protocols.io to enhance the reproducibility of your results. Protocols.io assigns your protocol its own identifier (DOI) so that it can be cited independently in the future. For instructions see: https://journals.plos.org/plosone/s/submission-guidelines#loc-laboratory-protocols. Additionally, PLOS One offers an option for publishing peer-reviewed Lab Protocol articles, which describe protocols hosted on protocols.io. Read more information on sharing protocols at https://plos.org/protocols?utm_medium=editorial-email&utm_source=authorletters&utm_campaign=protocols.

As the corresponding author, your ORCID iD is verified in the submission system and will appear in the published article. PLOS supports the use of ORCID, and we encourage all coauthors to register for an ORCID iD and use it as well. Please encourage your coauthors to verify their ORCID iD within the submission system before final acceptance, as unverified ORCID iDs will not appear in the published article. *Only*  the individual author can complete the verification step; PLOS staff *cannot*  verify ORCID iDs on behalf of authors.

We look forward to receiving your revised manuscript.

Kind regards,

Pavle Randjelovic, Ph.D.

Academic Editor

PLOS One

Journal Requirements:

1. If the reviewer comments include a recommendation to cite specific previously published works, please review and evaluate these publications to determine whether they are relevant and should be cited. There is no requirement to cite these works unless the editor has indicated otherwise.

2. Please review your reference list to ensure that it is complete and correct. If you have cited papers that have been retracted, please include the rationale for doing so in the manuscript text, or remove these references and replace them with relevant current references. Any changes to the reference list should be mentioned in the rebuttal letter that accompanies your revised manuscript. If you need to cite a retracted article, indicate the article’s retracted status in the References list and also include a citation and full reference for the retraction notice.

Additional Editor Comments:

The revised manuscript has addressed the major scientific and methodological concerns raised during peer review. The additional methodological details, clarification of the experimental procedures, expanded ethical statement, revised Data Availability Statement, and more cautious discussion have substantially strengthened the manuscript.

Before acceptance, the authors should address a few remaining editorial issues, including clarification of the auditory data presentation (left ear vs bilateral measurements where appropriate) and slightly moderating the wording of the conclusions regarding the therapeutic potential of MnTBAP.

[Note: HTML markup is below. Please do not edit.]

[NOTE: If reviewer comments were submitted as an attachment file, they will be attached to this email and accessible via the submission site. Please log into your account, locate the manuscript record, and check for the action link "View Attachments". If this link does not appear, there are no attachment files.]

To ensure your figures meet our technical requirements, please review our figure guidelines: https://journals.plos.org/plosone/s/figures

You may also use PLOS’s free figure tool, NAAS, to help you prepare publication quality figures: https://journals.plos.org/plosone/s/figures#loc-tools-for-figure-preparation.

NAAS will assess whether your figures meet our technical requirements by comparing each figure against our figure specifications.

# Author comment: Author response to Decision Letter 2

**DOI:** 10.1371/journal.pone.0351911.r004

2 Sep 2026

We have clarified in the methods section under statistical analysis that we used the data generated from the left ear and did not include bilateral measurements in the analysis. We have also moderated the wording of the conclusions.

We request to consider the revised manuscript for publication.

Attachment Submitted filename: Response to Reviewers.docx

# Editor report: Decision Letter 2

**DOI:** 10.1371/journal.pone.0351911.r005

**Authors:** Pavle Randjelovic

3 Sep 2026

Otoprotective effect of MnTBAP in cisplatin-induced hearing loss

PONE-D-26-27420R2

Dear Dr. Jamesdaniel,

We’re pleased to inform you that your manuscript has been judged scientifically suitable for publication and will be formally accepted for publication once it meets all outstanding technical requirements.

Within one week, you’ll receive an e-mail detailing the required amendments. When these have been addressed, you’ll receive a formal acceptance letter and your manuscript will be scheduled for publication.

An invoice will be generated when your article is formally accepted. Please note, if your institution has a publishing partnership with PLOS and your article meets the relevant criteria, all or part of your publication costs will be covered. Please make sure your user information is up-to-date by logging into Editorial Manager at Editorial Manager® and clicking the ‘Update My Information' link at the top of the page. For questions related to billing, please contact billing support.

If your institution or institutions have a press office, please notify them about your upcoming paper to help maximize its impact. If they’ll be preparing press materials, please inform our press team as soon as possible -- no later than 48 hours after receiving the formal acceptance. Your manuscript will remain under strict press embargo until 2 pm Eastern Time on the date of publication. For more information, please contact onepress@plos.org.

Kind regards,

Pavle Randjelovic, Ph.D.

Academic Editor

PLOS One

Additional Editor Comments (optional):

The authors have adequately addressed the remaining editorial issues raised in the previous decision letter, including clarification of the auditory data analysis and moderation of the conclusions regarding the therapeutic potential of MnTBAP. The revised manuscript is now suitable for publication in PLOS ONE. I therefore recommend acceptance.

Reviewers' comments:

# Editor report: Acceptance letter

**DOI:** 10.1371/journal.pone.0351911.r006

**Authors:** Pavle Randjelovic

PONE-D-26-27420R2

PLOS One

Dear Dr. Jamesdaniel,

I'm pleased to inform you that your manuscript has been deemed suitable for publication in PLOS One. Congratulations! Your manuscript is now being handed over to our production team.

At this stage, our production department will prepare your paper for publication. This includes ensuring the following:

* All references, tables, and figures are properly cited

* All relevant supporting information is included in the manuscript submission,

* There are no issues that prevent the paper from being properly typeset

You will receive further instructions from the production team, including instructions on how to review your proof when it is ready. Please keep in mind that we are working through a large volume of accepted articles, so please give us a few days to review your paper and let you know the next and final steps.

Lastly, if your institution or institutions have a press office, please let them know about your upcoming paper now to help maximize its impact. If they'll be preparing press materials, please inform our press team within the next 48 hours. Your manuscript will remain under strict press embargo until 2 pm Eastern Time on the date of publication. For more information, please contact onepress@plos.org.

You will receive an invoice from PLOS for your publication fee after your manuscript has reached the completed accept phase. If you receive an email requesting payment before acceptance or for any other service, this may be a phishing scheme. Learn how to identify phishing emails and protect your accounts at https://explore.plos.org/phishing.

If we can help with anything else, please email us at customercare@plos.org.

Thank you for submitting your work to PLOS One and supporting open access.

Kind regards,

PLOS One Editorial Office Staff

on behalf of

Dr. Pavle Randjelovic

Academic Editor

PLOS One
